# Response to ‘using social media to support small group learning’

**DOI:** 10.1186/s12909-019-1458-5

**Published:** 2019-01-29

**Authors:** Omkar Rajesh Deodhar, Jai Mathur

**Affiliations:** St George’s University of London, Cranmer Terrace, London, SW17 0RE UK

**Keywords:** Social media, Education, Communication

## Abstract

This letter serves to respond to the article from the point of view of medical students. It offers critical perspectives on how to take the research further in certain areas and alternatives that can be investigated. It also reinforces conclusions made by the article, as well as bringing to light new ideas, advantages, and limitations of the findings of the study.

Dear Editor,

We appreciate Cole et al.’s article regarding the use of social media in small group teaching and would like to thank them for their innovative research. We agree that ‘social media can be used constructively to complement CBL’ (Case Based Learning) [1]. Social media is used at SGUL (St George’s, University of London) to facilitate communication between members of the same CBL group; for example, several CBL groups have WhatsApp group chats which they use to communicate with each other. Not only that, but there are larger group chats that are used by entire year groups. There are also several Facebook pages which allow communication between the different year groups as well as allowing all students with Facebook, that is, the vast majority, to access a variety of different opportunities that are available to them ranging from extra-curricular to CV-boosting (Curriculum Vitae) activities to revision sessions hosted by older medical students. However, although SGUL does use social media for promotion and outreach, it does not currently use social media for learning. We believe that this may change given time, due to the increasing prevalence of social media use generally. This can be seen, as use of the internet for social networking rose from 45% in 2011, to 66% in 2017 [2].

Social media is now a part of modern life [3]. We feel it has a lot of potential to be used in medical learning. As shown by Cole et al.’s study, there are benefits for students [1]. Social media is an excellent platform for sharing ideas and experiences with one another, as well as resources and records as well. Not only that, but because social media is and will be so heavily intertwined in the lives of most students currently, and in the future, there is already a familiarity with the social media platforms. This eradicates the need to teach students how to use a new piece of software, such as those implemented in the study. For example, in the study, the students used ‘Scoop.it’ and a ‘wiki’ to aid their CBL learning and they reported that not everyone in the group understood how to use ‘Scoop.it’, and that the wiki was difficult to use [1]. As most students use Facebook, they are familiar with the website and how to use it. This eliminates certain logistical problems such as developing a new online learning platform to enable students to collaborate and communicate efficiently. Instead, they can use one that is already in place. Some input may be required, such as creating a page on which students can share documents and communicate with each other.

However, there are challenges that arise as well with the use social media, specifically regarding the impact that social media has upon the lives of students. The lives of doctors have been altered by social media [4], thus, the lives of students will have as well. As such, students need to be aware of the dangers of social media and need to know how to manage it effectively and safely. For example, the social media app, Instagram was reported as the ‘worst for young mental health’, in an article written by the BBC (British Broadcasting Corporation) [5]. Education regarding the responsible use of social media may be necessary for students. This is already a reality, as we have attended lectures hosted by SGUL about professionalism, and within these lectures, social media played a pivotal role. As well as this problem, the students in the study reported that sometimes their personal and professional lives would mix due to the use of social media and that the resultant distractions were problematic [1]. Furthermore, if the tutors are asked to engage with students using social media, the boundaries between the student-tutor relationships could blur, which could potentially lead to detrimental effects on both the student and the tutor. Perhaps, in this case, Facebook may not be the best social media platform to aid student’s learning. Instead, another social networking site could be used, for example, LinkedIn, a more professional social networking app.

In conclusion, there are both positives and negatives to the use of social media in the classroom. We agree with the conclusions that the authors have come to and believe that social media has a lot of potential to be used not just by medical students, but also possibly by all students across the globe. We propose investigating the use of other social media platforms such as LinkedIn or possibly developing another social media platform designed specifically for education within medicine. Social media is a powerful tool that has the capacity to revolutionise education due to its vast scope and global connectivity. However, as with all revolutionary ideas, there are risks, as the article rightly highlights. The way in which social media is integrated into the education system needs to be handled with great care, to ensure that it is not misused. If used correctly, however, it could lead to better education for all, which is a goal worth striving for.

## Abbreviations

BBC: British Broadcasting Corporation
CBL: Case Based Learning
CV: Curriculum Vitae
SGUL: St. George’s, University of London
